# Tracing modern breeding introgressions in European potato

**DOI:** 10.1007/s00122-025-05143-z

**Published:** 2026-02-17

**Authors:** Craig I. Dent, Lisa C. Baus, Sergio Tusso, Klaus J. Dehmer, Ronald C. B. Hutten, Herman J. van Eck, Korbinian Schneeberger

**Affiliations:** 1 Department of Chromosome Biology, Max Planck Institute for Plant Breeding Research, Cologne, Germany; 2 Cluster of Excellence on Plant Sciences, Heinrich-Heine University, Düsseldorf, Germany; 3 Faculty of Biology, LMU Munich, Planegg-Martinsried, Germany; 4 Leibniz Institute of Plant Genetics and Crop Plant Research (IPK), Gross Luesewitz, Germany; 5 Plant Breeding, Wageningen UR, Wageningen, The Netherlands

## Abstract

The European potato germplasm originated from a few founding genotypes, and its narrow genetic base has since been broadened through introgressions from wild relatives. We combined pedigree records and genome-wide SNP data to trace the origin, spread, and contributing ancestors of modern breeding introgressions in Europe. We first used a curated pedigree database to identify the Major Contributing Ancestors (MCAs) of 1209 varieties from the European Common Catalogue, revealing influential cultivars such as Katahdin, Saskia, and Agria as the top contributors to the modern European gene pool. Building on this framework, we developed a modified MCA approach that uses SNP alleles to trace the spread of haplotypes that were introduced into the European germplasm after 1945; two of which now occur in half of European varieties. Using the pedigree database to find the origin of these modern introgressions, we traced key contributions from *S. vernei*, *S. demissum*, and *S. tuberosum* Group Andigena clone CPC 1673. We observed multiple distinct haplotypes of the R3a/b late blight resistance introgression on chromosome 11. Additionally, we generated a genome assembly of *S. demissum* to validate a single sub-genome origin of the R3a/b introgression. We also traced a putatively starch-associated introgression derived from *S. vernei*. Our framework links historical breeding records with genomic data, revealing the legacy of modern introgression breeding in the European germplasm.

**Supplementary Information:**

The online version contains supplementary material available at 10.1007/s00122-025-05143-z.

## Introduction

The potato (*Solanum tuberosum* Group Tuberosum) was domesticated in South America and arrived in Europe through a limited number of introductions. There it encountered further bottlenecks, such as the adaptation to European environments and repeated late blight epidemics, which led to the loss of susceptible material (Glendinning 1983; Gutaker et al. 2019). As a result, early European potato breeding began from a shallow genetic base: a pool of varieties containing high sequence diversity but low haplotype diversity (Sun et al. 2025).

Systematic breeding records appear after 1850 (van Berloo et al. 2007), but the adoption of controlled crossing was gradual. For instance, Salaman (1926) listed 89 widely grown British varieties, of which only 24 were the offspring of intentional crosses; this reflects the slow transition between open pollination and controlled breeding in Europe. Since then, there has been limited sexual recombination (an average seven-year generation time between varieties), which means that founding haplotypes have been transmitted largely intact, persisting in long haploblocks across generations (Bradshaw 2009; Sun et al. 2025; Vos et al. 2017). This history of admixture in Europe, followed by a limited number of sexual generations, helps to explain the weak population structure observed today in cultivated potato. Potato shows only a subtle separation of subpopulations, probably driven by founder effects: for example, many processing varieties descend from the German variety Agria (D’hoop et al. 2010; Tuttle et al. 2024; Vos et al. 2017).

To understand how this narrow genetic base shaped modern varieties, methods have been developed to identify which founding varieties contributed most to a pedigree, so-called ‘Major Contributing Ancestors’ (MCAs). For example, Love (1999) analysed a set of 44 prominent North American varieties and leveraged pedigree information to identify 12 MCAs, ranking them by their genetic contribution to the prominent varieties (Lansari et al. 1994; Sjulin & Dale 1987). Since then, the Online Potato Database (van Berloo et al. 2007) has enabled the direct counting of contributions to commercial potato varieties (Li et al. 2018), and the identification of the most frequent ancestors of global sub-populations (based on a worldwide panel of 231 varieties; Deperi et al. 2018). To our knowledge, the identification of the Major Contributing Ancestors of the European gene pool has not been carried out using these pedigree resources.

Beyond the contributions of historical cultivars, modern breeding has further reshaped the European gene pool through targeted introgressions from wild relatives. In the second half of the twentieth century, breeders increasingly focussed on introgression breeding, the deliberate introduction of traits from wild relatives through backcrossing (Bradshaw 2022). This led to the introduction of new haplotypes into the European gene pool (Bradshaw 2022; Vos et al. 2015), which also persist as large haploblocks (Vos et al. 2017). Introgression breeding typically targeted disease resistance against late blight, viruses, and nematodes (Bradshaw 2022). For example, the *H1* locus at the end of chromosome 5, which confers resistance to Potato Cyst Nematode (PCN), was introgressed from the *S. tuberosum* Group Andigena clone CPC 1673 (Ellenby 1952; Gebhardt et al. 1993).

Another widely used introgression donor was the hexaploid *S. demissum*, which notably contributed the resistance locus *R3a/b* at the end of chromosome 11, conferring resistance to late blight (Huang et al. 2004). *S. demissum* introgressions in Europe came from diverse material: resistance was originally identified in a hybrid of *S. demissum* and *S. tuberosum* (*S.* × *edinense*), and early resistance breeding in Germany used similar material, namely the clone of uncertain origin ‘Edinense Fraglich’ (EF) (Müller 1951). In later introgressions, *S. demissum* itself was used, notably in Scotland by W. Black via a bridging cross with *S. rybinii* (Black 1946), and both Russian- and American- collected clones were used at the Dutch breeding company CEBECO in the 1940s (Mastenbroek 1966).

Despite a diversity of donors, introgressions rise to high frequency not through the donors themselves, but through the repeated use of breeding lines or prominent varieties in which the introgression has been isolated. For example, the breeding line VTN 62–33-3, developed in a pre-breeding programme by the Dutch Foundation for Plant Breeding (*Stichting voor Plantenveredeling*) (van Berloo et al. 2007). VTN 62–33-3 has three sources of *S. vernei* in its recent pedigree and has been identified as the most frequent source of introgressed alleles appearing after 1945, accounting for 12.7% of introgressed SNPs (Vos et al. 2015). Notable loci introduced by this variety include the GPA5 locus on chromosome 5, conferring *Globodera pallida* nematode resistance (Rouppe Van Der Voort et al. 2000; Van Eck et al. 2017). Some of the SNPs first observed in VTN 62–33-3 have risen to high allele frequencies amongst starch varieties, likely due to a founder effect (Vos et al. 2015).

Whilst the genomic footprint of wild introgressions is increasingly well-documented (Bao et al. 2022; Hoopes et al. 2022; Sun et al. 2025; Vos et al. 2015), how individual introgression haplotypes were transmitted through breeding lineages, and how these contributions intersect with historical founder effects, remains unresolved.

Here, we address these gaps by first identifying the Major Contributing Ancestors (MCAs) of 1,209 European varieties using a curated pedigree database. We then extend this framework to incorporate genome-wide SNP data, applying a modified MCA algorithm to trace the transmission of individual introgressions from wild potato species into the European germplasm. This approach enables us to link modern genomic data with historical breeding records, revealing which donor haplotypes were successfully integrated, the lineages through which they spread, and how their frequencies changed over time.

## Results

### Curation of the European potato pedigree

We collated pedigree information from the Wageningen University & Research Potato Pedigree (van Berloo et al. 2007) and the Gross Lusewitz Potato Collections at the Leibniz Institute of Plant Genetics and Crop Plant Research (IPK) (Klaus J. Dehmer, personal communication). We manually curated these records and identified 12,041 named varieties (including 770 varieties with synonymous names; Supplementary Data 1).

Records of crosses between defined potato varieties in Europe begin in earnest in the 1870s. However, the pedigree information was more complete for recent varieties; we saw a steep jump in missing parentage in records prior to 1900 (Supplementary Table S1).

The curated pedigree primarily consisted of European breeding records. Of the 4,852 varieties which had a recorded country of origin, 83.5% (4,050/4,852) originated in European countries, followed by 9.4% (457/4,852) for North America and 7.1% (345/4,852) for the rest of the world. Within Europe, the curated pedigree contained mostly Dutch (32.2%), German (22.8%), and British (9.5%) varieties. We observed that 95% of recent ancestors (parents or grandparents) of European varieties released after 1900 were themselves European varieties, suggesting limited exchange between continents in the modern breeding history of European potato.

Within Europe, we saw evidence for a systematic preference towards varieties having recent ancestors (parents or grandparents) from the same country, with 65% of the recent ancestors of Dutch and British varieties originating from the same country. In German varieties released after 1900, 83.7% of their recent ancestors were themselves German varieties (1,602/1,915 ancestors; Supplementary Table S2).

### The major contributing ancestors of European varieties

Before investigating the contributions of introgressions to the European gene pool, it was important to understand what are the Major Contributing Ancestors of the European gene pool per se*.* To represent European potato varieties, we took the list of potato varieties in the European Commission Common Catalogue of Agricultural Plant Species (Common Catalogue of varieties of agricultural plant species 2021), which lists all cultivars to be marketed in the European Union. We identified 1,209 out of 1,706 of these varieties present in the curated pedigree (70.9%; Supplementary Table S3). These varieties were almost exclusively of European origin (98.1%) and were typically, but not always, modern varieties; spanning the year 1850 (Pink Fir Apple) to the year 2020 (26 varieties; Supplementary Table S3). Tracing the pedigree records of these varieties, we observed records of 2,996 ancestors.

**Fig. 1:**
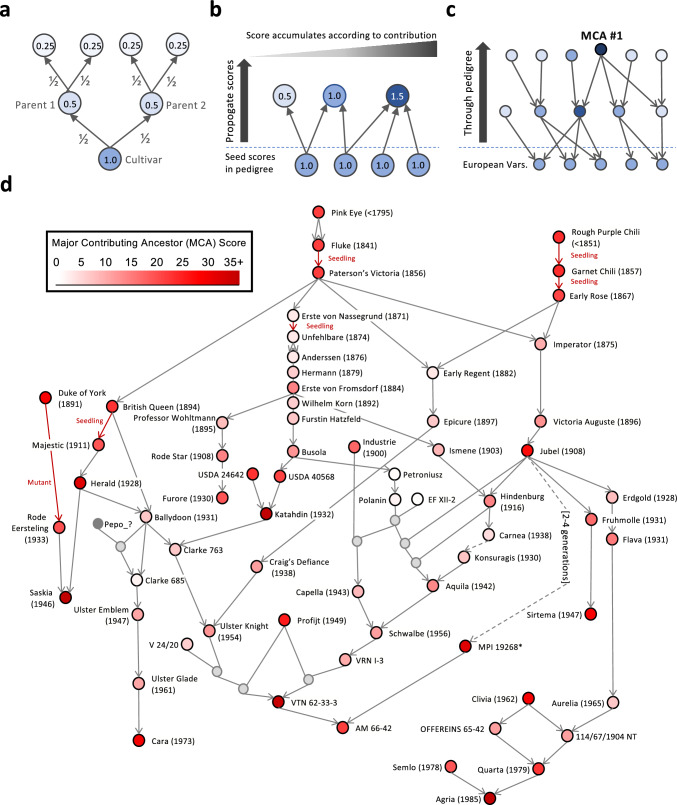
Relationships between the top 25 Major Contributing Ancestors (MCAs) of European varieties. A brief methodological overview: (**a**) Genetic contribution to a variety is calculated by passing half of its score up to its parents. (**b**) Varieties of interest are seeded with a score of 1. Ancestral varieties receive half of the score of all their children. If an ancestor has made a large contribution to score-seeded varieties, then it will accumulate a large score. (**c**) Scores are seeded into representative European varieties in order to identify their highest ranking Most Contributing Ancestors (MCAs). (**d**) Interrelationships of the top 25 MCAs. Node colours indicate the contribution score of a variety. Light grey nodes are unnamed hybrids. Dark grey nodes indicate parentage that could not be unambiguously identified in the pedigree. Grey arrows indicate direction of inheritance. Red arrows indicate descent through mutation or where records only indicate ‘seedling’ of a cultivar. Varieties are arranged by year of release (when known) from top to bottom. *The pedigree contains two accounts of the lineage between MPI 19268 and Jubel, varying from 2 to 4 generations (Supplementary Fig. 2)

We then asked which of these ancestors made the largest contributions to European potato. We adapted the methods of Sjulin and Dale (1987) to calculate the Major Contributing Ancestors (MCAs) of the European varieties (Table 1, Fig. 1a–c). Briefly: each European variety is assigned a score of 1, and half of that score is passed to each parent through the pedigree. Scores typically decay over generations, but varieties appearing in the ancestry of many European varieties accumulate higher scores, identifying them as major contributing ancestors. In total, there were 437 ancestors with a score greater than 2 (equivalent to having directly parented more than four European varieties; Supplementary Table S4). Katahdin was the greatest contributor to European varieties with a score of 38.28 (Table 1). Katahdin appeared in the ancestry of 57.5% of European varieties (696/1,209), via 3,277 unique walks through the pedigree (an average of 2.7 times per variety).

**Table 1:** The top 25 Major Contributing Ancestors of European potato. “# Offspring” is the number of immediate descendants in the pedigree. *MPI 19268 (Rank 5) is tagged as MPI 19268_? elsewhere in the analysis because of unresolved conflicting data regarding its parentage, however this does not affect its scoring as an MCA

Rank	Cultivar	Year	Origin	# Offspring	Score
1	Katahdin	1932	USA	220	38.28
2	Saskia	1946	Netherlands	48	37.02
3	Agria	1985	Germany	100	36.93
4	VTN 62–33-3	1962	Netherlands	61	34.47
5	MPI 19268*	< 1961	Germany	22	30.87
6	Herald	1928	Great Britain	14	30.05
7	Cara	1973	Ireland	60	29.63
8	Duke of York	1891	Great Britain	30	29.30
9	Clivia	1962	Germany	21	28.83
10	Sirtema	1947	Netherlands	34	28.47
11	Jubel	1908	Germany	66	26.67
12	Profijt	1949	Netherlands	14	25.15
13	Garnet Chili	1857	USA	7	24.03
13	Rough Purple Chili	< 1851	Chilean landrace	1	24.03
15	USDA 24642	< 1932	USA	10	23.12
16	British Queen	1894	Great Britain	6	23.08
17	Quarta	1979	Germany	14	22.71
18	AM 66–42	1966	Netherlands	47	21.86
19	Fluke	1841	Great Britain	2	21.80
19	Paterson’s Victoria	1856	Great Britain	18	21.80
19	Pink Eye	< 1795	Great Britain	1	21.80
22	Early Rose	1867	USA	69	21.43
23	USDA 40568	< 1932	USA	4	20.36
24	Furore	1930	Netherlands	27	19.63
25	Semlo	1978	Germany	5	19.59

We investigated the relationships between the top 25 MCAs and found considerable interrelationships across generations (Fig. 1d). For example, AM 66–42 (Rank 18) is a descendant of a cross between VTN 62–33-3 (Rank 4) and MPI 19268 (Rank 5), and has contributions from Paterson’s Victoria (Rank 19) via 11 unique paths in the pedigree (ranging from seven to eighteen generations) which include German, US, British, and Dutch breeding programmes (Fig. 1d).

We also saw accumulation of MCA scores at putative donor sources of wild introgressions. For example, the summed score of all 13 clearly labelled introgression sources of *S. vernei* in the pedigree was 21.0. This would hypothetically rank *S. vernei* amongst the top 25 MCAs of European potato varieties (Supplementary Table S4).

### The major contributing ancestors of modern introgressions

We then adapted our MCA methodology to trace the contributions of introgressions from wild relatives. First, we identified haplotypes that were likely introduced to the European gene pool through modern introgression breeding. To do this, we used SNP calls from the 20 K Infinium SNP array (Vos et al. 2015, 2017), genotyped on 886 samples. In this dataset, we identified 365 samples which could be assigned to varieties in the pedigree database (Supplementary Table S5). Following the lead of Vos et al., we further identified 47 historical varieties that were released prior to the year 1945. We identified 3,449 SNPs which were homozygous in these 47 historical varieties but variable across the remaining 318 varieties, suggesting that these alleles were introduced by modern breeding into the European gene pool (Supplementary Data S2).

We then clustered these SNPs by allele dosage and identified 127 clusters of co-occurring SNPs, with a minimum allele frequency of 0.3% (Supplementary Table S6; Supplementary Figs. 3–14). Interestingly, even though we did not use chromosomal position during the clustering of the SNPs, the SNPs of most clusters were located in well-defined regions of the genome, likely outlining the borders of the underlying introgressions. These clusters contained anywhere from 5 to 46 SNPs and spanned from kilobases up to entire chromosomes (1.3 kb to 83.7 Mb; Supplementary Table S6). We found three clusters which were outstandingly frequent amongst the 196 European varieties for which we had genotypic data. For these three clusters, we investigated their Major Contributing Ancestors.

**Fig. 2:**
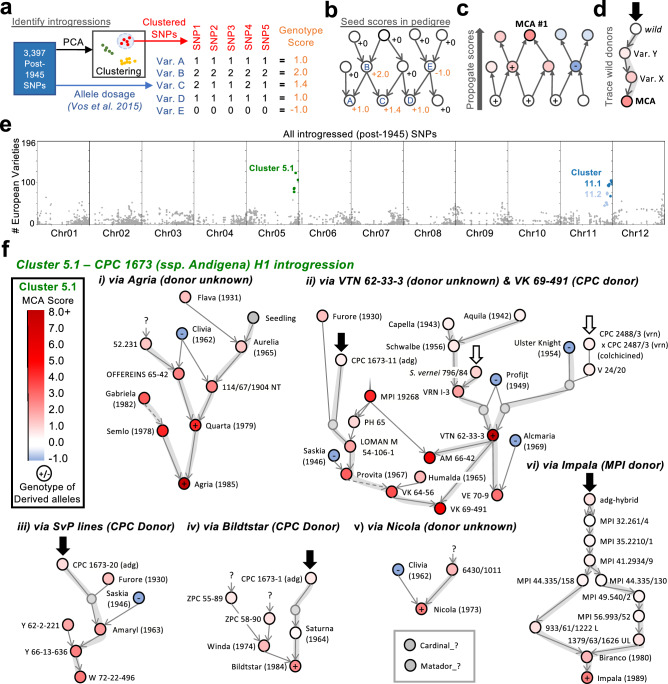
Identification of introgressed haplotypes and their Major Contributing Ancestors (MCAs). (**a**) Schematic overview of SNP clustering: SNPs that were homozygous in varieties before 1945 were clustered based on the dosage of their derived alleles. Genotype scores for each variety were assigned as an average of the derived allele dosage. (**b**) Scores were then seeded in the pedigree: varieties carrying derived alleles (Variety A–D) received positive genotype scores, whilst those lacking them (Variety E) received a genotype score of − 1.0. (**c**) Scores are propagated up the pedigree, with the exception that scores cannot propagate through varieties which received a genotype score of − 1.0 (e.g. middle right node). (**d**) Once MCAs have been identified, pedigree records can be traced further back to identify the likely introgression donors. (**e**) Visualisation of the frequency of post-1945 SNPs reveals three clusters of interest (coloured dots), which represent introgressions that occur in European varieties at a higher allele frequency than other introgressed clusters (grey dots). (**f**) Pedigree reconstructions illustrate the different routes through which CPC 1673 contributed to European germplasm: (i) via Agria, (ii) via VTN 62–33-3 and VK 69–491, (iii) via *Stichting voor Plantenveredeling* breeding lines, (iv) via Bildtstar, (v) via Nicola, and (vi) via Impala. Arrows indicate inheritance through the pedigree. Grey nodes are unnamed hybrids. A plus/minus symbol in the node indicates a non-zero or zero allele dosage, respectively. Dashed lines indicate conflicting records of parentage in the pedigree. Black/white arrows indicate potential introgression donors. Grey paths highlight possible paths between MCAs and their introgression donors. Abbreviations: adg = *S. tuberosum* Group Andigena, vrn = *S. vernei*

To trace the origin and ancestry of these introgressions, we adapted our MCA approach to work with SNP data. For the 365 genotyped varieties in the pedigree (Supplementary Table S5), we assigned them a score based on their dosage of derived alleles from a given SNP cluster (Fig. 2a). To prevent contribution scores from simply accumulating at MCAs per se*,* we assigned negative scores to genotyped varieties completely lacking the derived alleles (Fig. 2b). We then propagated these scores upward through the pedigree to identify Major Contributing Ancestors as before (Fig. 2c). This allowed us to highlight the lineages through which the introgression likely entered the gene pool (Fig. 2d).

In the following, we present the details of this analysis for the two introgressions with the highest allele frequency in European potatoes:

**Cluster 5.1** was observed on the south arm of chromosome 5, it spanned 4.5 Mb and was defined by 7 SNPs. Almost half of European varieties (51%) contained alleles from this cluster (Fig. 2e; Supplementary Table S6); however, we observed a variety of dosages of the introgressed alleles (Supplementary Fig. 17; Supplementary Table S14). These alleles matched a reported high-frequency introgression in linkage with the *H1* locus conferring Potato Cyst Nematode (PCN) resistance (Gebhardt et al. 1993; Turner 1989; Vos et al. 2015). We performed the modified MCA analysis and identified the top 25 MCAs of the introgression and six independent pedigree lineages contributing the clustered alleles (Fig. 2f; Supplementary Fig. 16, Supplementary Table S9). Using the pedigree records, we were able to trace the MCAs back to the widely used *S. tuberosum* Group Andigena clone CPC 1673 in four out of six lineages (Fig. 2f i-vi). We expected multiple lineages because the donor clone CPC 1673 was recorded in the pedigree under multiple different aliases. For the sixth lineage, containing the variety Impala, we were able to trace back to a Group Andigena ancestor ‘adg-hybrid’ (Fig. 2f vi); however, it appears that this introgression (circa 1932) would predate the discovery of the original CPC 1673 clone (circa 1952); thus, the introgression likely reached Impala through an unrecorded lineage (Supplementary Fig. 16).

**Fig. 3:**
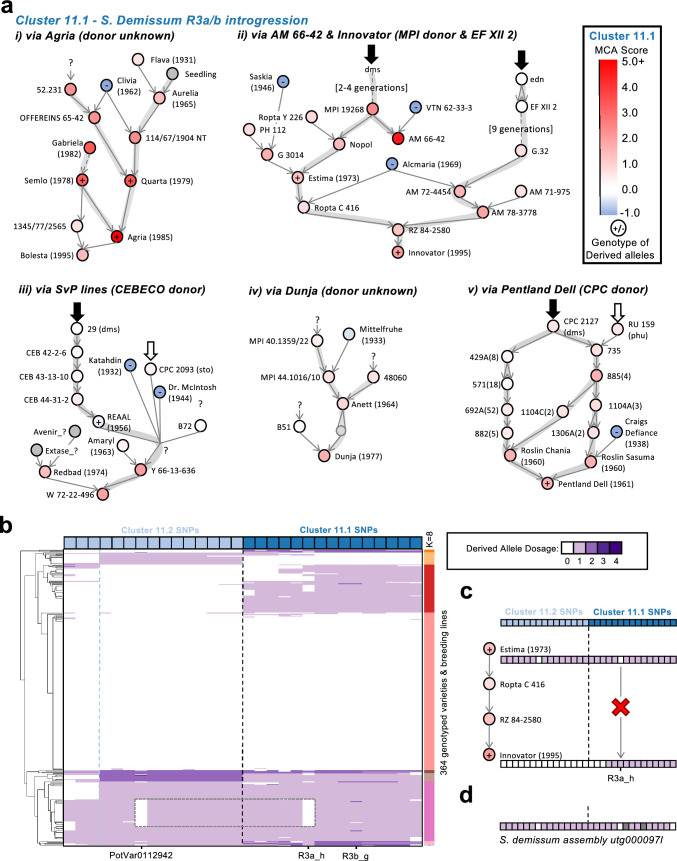
The Major Contributing Ancestors (MCAs) of the R3a/b introgression from *S. demissum* (**a**) Pedigree reconstructions illustrate the different routes through which *S. demissum* contributed the R3a/b locus to the European germplasm: (i) via Agria, (ii) via Innovator, (iii) via *Stichting voor Plantenveredeling* (SvP) breeding lines, (iv) via Dunja, (v) via Pentland Dell. Arrows indicate inheritance through the pedigree. Small grey nodes are unnamed hybrids. Dark grey nodes are cultivars with unresolved duplicate names. Node colour indicates its MCA score (negative values are capped at -1.0). A plus/minus symbol in the node indicates a non-zero or zero allele dosage where known. Dashed lines indicate conflicting records of parentage in the pedigree. Black/white arrows indicate potential introgression donors. Grey paths highlight possible paths between MCAs and their introgression donors. Abbreviations: dms = *S. demissum*, phu = *S. tuberosum* Group Phureja, sto = *S. stoloniferum,* chc = *S. chacoense,* edn = *S.* × *edinense* (a natural hybrid between *S. demissum and S. tuberosum*), (**b**) Haplotype clustering of SNPs from clusters 11.2 and 11.1. The purple heatmap along the x-axis shows the dosage of the derived allele for each SNP. Genotyped varieties are arranged along the y-axis. A dendrogram on the far left shows clustering based on allele dosage of SNPs. The black dashed line (middle) shows the putative recombination breakpoint between the clusters. The blue dashed line (left) shows another potential recombination breakpoint within cluster 11.2. The dashed grey box (bottom) highlights the linkage between two SNPs (PotVar0112942 and R3a_h) which define a secondary haplotype. (**c**) Differing haplotypes (marked by the R3a_h SNP) in Estima and its descendant Innovator (MCAs featured in panel a-ii) apparently rule out inheritance of the *R3a/b* locus via this pedigree lineage. (**d**) The presence/absence of SNPs along a single contig of the ‘El Desierto’ (*S. demissum*) draft genome assembly. Purple boxes indicate that the allele was present. White boxes indicate that the allele was absent. Grey boxes indicate that the contig was not aligned to the reference genome at this position, and so the allele could not be called

We were unable to identify the putatively introgressed alleles in publicly available sequencing data of samples of the *S. tuberosum* Group Andigena (Supplementary Table S12), or in two scaffold-level genome assemblies of Andigena (ADG1-CIP 700921 and ADG2-CIP 702853; Supplementary Table S13) (Kyriakidou et al., 2020), probably owing to the rarity of this haplotype, even amongst the clones of the original collection (Ellenby 1952).

**Fig. 4:**
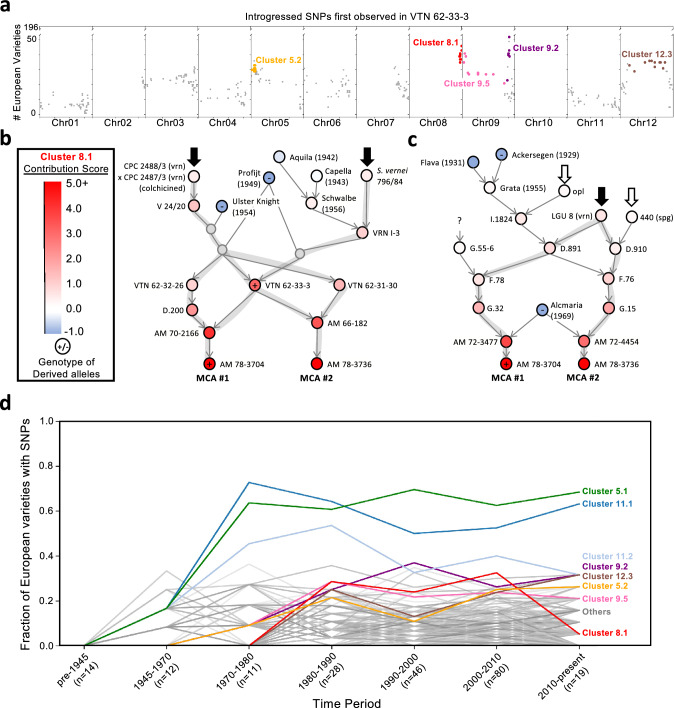
MCA analysis of SNPs first observed in VTN 62–33–3 reveals multiple breeding targets. (**a**) Alleles first observed in VTN 62–33–3 produce at least five clusters of interest (Coloured points), which occur in European varieties more often than other introgressed alleles from the same variety (grey points). Cluster 8.1 SNPs are all located at the end of the long arm of chromosome 8 (red points). (**b**) AM breeding lines from the *Stitching voor Plantenveredeling* are identified as the top MCAs; both sharing VTN 62–33–3 as an ancestor. Subgraphs of the pedigree are shown, grey arrows indicate inheritance through the pedigree. Grey nodes are unnamed hybrids. (**c**) Although these SNPs were first observed in VTN 62–33–3, there are other potential introgression donors (black and white block arrows). Abbreviations: vrn = *S. vernei,* opl = *S.oplocense,* spg = *S.spegazzinii.* (**d**) Occurrences of SNP Clusters in European varieties over time. Each line is an individual cluster of SNPs which are only observed after 1945. The x-axis shows time periods, and how many of the genotyped European varieties were released within that period. The y-axis shows how many European varieties contain at least one of the clustered SNPs

**Cluster 11.1** occurred on the south arm of chromosome 11, and spanned 3.9 Mb. It was defined by 15 SNPs, which occurred in 49% of European varieties on average (Fig. 2e; Supplementary Table S6). The introgressed alleles included markers for the *R3a/b* locus, which was derived from *S. demissum* (Vos et al. 2015) and confers resistance to late blight (Huang et al. 2004*)*. Applying the modified MCA analysis, we identified the top 25 MCAs of these alleles and observed six pedigree lineages (Fig. 3a; Supplementary Fig. 18; Supplementary Table S9).

The highest contributor of Cluster 11.1 derived alleles was again Agria (Fig. 3a i), and other top contributors included: Dutch breeding lines from the *Stichting voor Plantenveredeling* such as AM 66–42, and the commercial varieties Innovator, Estima, and Pentland Dell (the latter a product of the Scottish Plant Breeding Research Station; Fig. 3a i-v). Tracing up the pedigree from these MCAs, we identified four different introgression donors from *S. demissum* and *S.* × *edinense* (a hybrid of *S. demissum* and *S. tuberosum*) (Fig. 3a i-v; Supplementary Fig. 18–19). Putative introgression donors included: one of several *S.demissum* clones used by the Max Planck Institute of Plant Breeding Research (Fig. 3a ii), the clone EF XII 2 (derived from the German ‘Edinense Fraglich’ clone), which gave rise to W-varieties widely used for late blight resistance (Müller 1951) (Fig. 3a ii), the clone 29 (*S. demissum*) from the Plant Breeding Station of Cebeco, which has its origins in either Russian or American collections in the 1920s and 1930s (Mastenbroek 1966) (Fig. 3 a iii), and CPC 2127, the *S. demissum* clone used by Black at the Scottish Plant Breeding Station (Fig. 3 a v).

We also observed a second cluster on Chromosome 11 (**Cluster 11.2**), which spanned the 2.5 Mb immediately upstream of cluster 11.1, but had an overall lower occurrence in European varieties (Fig. 2b; Supplementary Fig. 13). It shared many of its top MCAs with cluster 11.1 (Supplementary Table S9), suggesting a similar origin. We investigated the panel of genotyped varieties and saw clustering according to the dosage of alleles from these two clusters (Fig. 3b), indicating that the alleles from either cluster often segregate independently. However, the majority of varieties with alleles from one cluster contained alleles from the other cluster as well, indicating a larger introgression (represented by both clusters, 7.1 Mb), which had recombined into two blocks (Fig. 3b; Supplementary Table S15). Within varieties carrying the larger 7.1 Mb introgression, there was unexpected, but striking evidence for two highly similar but still distinct haplotypes. The two haplotypes could be distinguished by the absence of derived alleles of the SNPs PotVar0112942 and R3a_h (Fig. 3b; Supplementary Table S15). This strongly suggests that at least two different haplotypes of the *R3a/b* locus region were introgressed from *S. demissum* and/or *S.* × *edinense*, and were widely distributed in modern European breeding. With this insight, we were able to refine the ancestry of introgression cluster 11.1 and, for example, effectively rule out one of the possible introgression donors of the *R3a/b* locus to the variety Innovator (Fig. 3c).

To verify the origin of these cluster 11.1 and cluster 11.2 alleles, we selected an *S. demissum* sample from the Gross Lusewitz Potato Collections named ‘El Desierto’, which shares its name with a clone collected by Reddick in 1930 and used in German breeding as early as 1938 (Lehmann 1938; van Berloo et al. 2007). We generated a contig-level assembly using PacBio long reads and Oxford Nanopore ultra-long reads. Using whole-genome alignment to the DM reference genome, we saw three contigs at most positions, suggesting that the three sub-genomes of the hexaploid *S. demissum* have been assembled separately (Supplementary Table S13). In this assembly, we saw 25/28 of the introgressed alleles from clusters 11.1 and 11.2 (with two of the missing alleles being PotVar 0112942 and R3a_h; Fig. 3d). These SNPs all belonged to a single contig (Fig. 3d; Supplementary Table S13), suggesting that the late blight resistant *R3a/b* locus in *S. demissum* exists only on a single sub-genome.

We saw little evidence for these introgressed alleles in the group Andigena samples previously mentioned; however, we did see cluster 11.1 alleles on a single haplotype of the ‘improved’ Andigena cultivar Diacol Caprio (DC; Supplementary Table S13) (Reyes-Herrera et al., 2024), which likely has its origins in a European or North American (*S. tuberosum* Group Tuberosum) ancestor that already carried the introgression.

### The role of VTN 62–33–3 in modern introgressions

VTN 62–33–3 was previously found to be the largest contributor of alleles arising after 1945 (Vos et al. 2015). In line with this, we found that 29% of our high-confidence clusters (37/127) contained SNPs where the majority of derived alleles were first observed in VTN 62–33–3 (Supplementary Table S6). Given this, we looked more closely at alleles which were first observed in VTN 62–33–3. We identified additional clusters of interest which were frequent in European varieties, namely clusters: 5.2, 8.1, 9.2, 12.3 (Fig. 4a; Supplementary Table S6).

We considered which loci the VTN 62–33–3 SNP clusters might be tagging. Cluster 5.2 and 9.2 are presumably loci of Potato Cyst Nematode resistance, Grp1/Gpa5 and Gpa6 respectively (Rouppe Van Der Voort et al. 2000). Cluster 9.5 included a large part of Chromosome 9, including the centromere; and one of its SNPs (PotVar0011738) was only 8.2 kb upstream of a known marker of *S. vernei*-derived late blight resistance (STM1051) (Milbourne et al., 1998; Sørensen et al., 2006). However, cluster 12.3 (spanning the centromere of chromosome 12) and cluster 8.1(on the south arm of chromosome 8) did not have obvious explanations. Cluster 8.1 was of particular interest, given that we observed no other alleles derived from VTN 62–33–3 on the entirety of chromosome 8.

**Cluster 8.1** had the highest average allele frequency of the VTN-62–33-3-derived clusters (Fig. 3a; Supplementary Table S6), occurring in 20.1% of European varieties (39.7/197 varieties, averaged across all 10 SNPs) and was localised to a 2.7 Mb region (56.3–59.0 Mb). Applying our modified MCA analysis, we revealed a single pedigree subgraph, rooted by the top two MCA varieties, AM 78–3704 and AM 78–3736, both breeding lines of the Dutch *Stitching voor Plantenveredeling* (Fig. 4b-c; Supplementary Table S9; Supplementary Fig. 15). AM 78–3704 was genotyped with Cluster 8.1 SNPs on two haplotypes (Supplementary Table S9), whereas AM 78–3736 was not genotyped. These two top scoring contributors share three *S. vernei* ancestors including V 24/20 (a colchicined cross of two *Solanum vernei* accessions), *S. vernei* 796/84 (Fig. 4b), and LGU 8 (vrn) (Fig. 4c), mirroring a previous pedigree of the VTN 62–33-3-derived GPA5 haplotype (Van Eck et al. 2017), which suggests that both haplotypes were introgressed together.

Given that the identity of cluster 8.1 is unclear, we searched for markers of QTLs and QRLs that might explain its selection (Supplementary Table S6; Gebhardt, 2023). Given the role of VTN 62–33–3 as a founder of modern starch varieties, we especially looked for QTLs related to starch synthesis. Cluster 8.1 does overlap previously identified QTLs for starch production containing a beta amylase gene at 58.75 Mb on chromosome 8 (Soltu.DM.08G029750; Schönhals et al. 2017).

We compared the alleles of VTN-62–33-3-derived clusters (5.2, 9.2, 8.1) to eleven published *S. vernei* WGS samples, and saw that 70.9% of the clustered alleles were present in at least one of the samples (Supplementary Table S11). We also saw a marginally stronger signal for Cluster 8.1 alleles in the assembled genome of *S. vernei* compared to its closest relatives (Supplementary Table S13, Supplementary Fig. 20). Together, these provide moderate support that these introgressions indeed derive from *S. vernei* donors. However, we cannot rule out other sources of introgression.

### The frequency of modern introgressions over time

Combining genotypic and pedigree data allowed us to track how these introgressions spread through European varieties over time. Cluster 5.1 and 11.1 have both occurred in more than half of the assessed European cultivars released since 1970, which continues until the present day (Fig. 4d). This is more than twice the frequency of any other SNP clusters first appearing in the same cultivars on any other chromosome (Supplementary Table S6), suggesting continued selection of the underlying loci. Cluster 11.2 showed a downwards trend from 1980 onwards (Fig. 4d), perhaps no longer being selected since this fragment of the larger *S. demissum* introgression apparently does not contain the R3a/b locus.

As expected from the previously reported founder effect (Vos et al. 2015), we saw VTN-62–33-3-derived SNP clusters significantly more often amongst starch cultivars (released 2000–2010) than expected by chance (X > 7.9, p < 0.01; Supplementary Table S7). However, we saw a dramatic drop in the occurrence of Cluster 8.1 in varieties released since 2010 (Fig. 4). This is likely linked to the reduced number of starch cultivars amongst the post-2010 European varieties that we assessed, only one variety (Euroflora) belonged to the “starch” subpopulation, and this was the variety with Cluster 8.1 SNPs (Supplementary Table S7). However, the persistence of the other VTN-62–33-3-derived introgressions in this final time period does support the hypothesis that the cluster 8.1 introgression has some particular importance for starch potato breeding.

## Discussion

We have characterised the Major Contributing Ancestors (MCAs) in the European potato according to the European Commission Common Catalogue, which contains varieties that can be marketed in the European Union (EU). The apparent bias towards Dutch, German, and British varieties in our pedigree (64.5% of varieties) is not surprising given that only five countries in Northwest Europe (Germany, France, Netherlands, UK, Belgium, so-called “NWEC-05”) accounted for more than 60% of potato production in the EU in 2021 (Goffart et al. 2022). However, it is possible that breeding efforts in Eastern Europe will be underrepresented in our study.

It was not surprising to see the North American varieties amongst the European MCAs. Katahdin (top ranked in our analysis) was previously identified as the greatest genetic contributor to prominent North American cultivars (Love 1999), but also the most frequent direct parent of potato varieties released worldwide (considering 1841–2013; X. Li et al. 2018). The US-bred Rough Purple Chili → Garnet Chili → Early Rose lineage was also previously seen to be a major contributor to prominent North American and British cultivars (Glendinning 1983; Love 1999), and has been shown to be the lineage through which *Sli*-mediated self-compatibility entered the European gene pool (Clot et al., 2020).

The other historical lineage at the top of our MCA pedigree (Fig. 1d) was British (Fluke → Pink Eye → Paterson’s Victoria); and was predicted a century ago, as it was remarked by Salaman (1926) that “Indeed, it may be said that practically no potato today of any outstanding merit is without the blood – though far removed –of this variety.” Other more recent European MCAs we found to be specific to the European germplasm: For example, the 3nd-ranked MCA Agria, bred by Agrico and released in 1985, was so often used that it accounts for its own cluster in the population structure of cultivated potato in Europe (D’hoop et al. 2010; Vos et al. 2015). Whilst Agria was previously flagged as a frequent ancestor of potatoes worldwide (X. Li et al. 2018), that signal may be driven by use of the same European-heavy pedigree database that we used here. Importantly, our introgression analysis has shown that Agria is the major contributor of the two most frequent introgressions found in European potato, although this is not necessarily why it was so popular. Anecdotally, Agria has been preferred for its good General Combining Ability. The large contribution of VTN 62–33-3 (4th rank) and MPI 19268 (5th rank) are more likely due to the introgressions they contained. Beyond this: Saskia (2nd rank) was perhaps helped by its inclusion in an unnamed hybrid, Saskia × (CPC 1673–20 (adg) × Furore), presumably containing the H1 locus, which was used frequently in modern European breeding (Deperi et al. 2018). Further, Cara (7th rank) and Sirtema (10th rank) have perhaps been underappreciated for their contribution to modern European potato.

There are several limitations of pedigree-based analyses in potato. The first, and most obvious, is incomplete records. The second limitation comes from conflicting records. For instance, in Fig. 1 we were unable to resolve the relationship of two MCAs: whether Jubel contributed to MPI 19268 either over two generations (via Parnassia), or four (via the lineage of Erdgold → Flava → MPI 27.1294/85) (Supplementary Fig. 2). Thirdly, cultivars with duplicate names can often not be resolved. Duplicate naming issues persist into modern breeding collections (Chrominski et al. 2024), and it has been observed that only 87% to 91% of pedigree trios can be verified by genotypic data (Endelman et al. 2017; Spanoghe et al. 2024), suggesting that misnaming and/or mislabelling issues are widespread. We addressed these limits in our study by taking a conservative approach (not propagating MCA scores through nodes with known namespace issues) and using a large number of samples.

Applying the MCA analysis to clusters of introgressed SNPs also has some limitations. In the analysis of Cluster 8.1, we saw that further genotyping would be needed to resolve the contributions of three different *S. vernei* donors to the same breeding programme. Our clustering method based on co-occurrence may only cluster common SNPs and fail to cluster SNPs that are unique to one particular introgression event. The MCA scores can also be influenced by sampling bias in the selection of varieties to be genotyped and by the number of alleles from a cluster that a variety transmits to offspring. In the future, these issues could be mitigated by tracing only individual or closely linked SNPs.

The most frequently observed introgressed haplotype was Cluster 5.1, presumably containing the *H1* PCN resistance locus contributed via *S. tuberosum* Group Andigena clone CPC 1673. This widely used clone was suggested to explain a subpopulation amongst Argentinian cultivars (Deperi et al. 2018), and also explains why Furore shows up as a Major Contributing Ancestor of the SNP cluster, as Saskia × (CPC 1673–20 (adg) × Furore) and CPC 1673–11 (adg) × Furore were present in the ancestry of 53% of the individuals of one subpopulation of potato cultivars (Deperi et al. 2018). Interestingly, the 2nd-ranked MCA for cluster 5.1 was VTN 62–33-3, whose pedigree would apparently rule out any Group Andigena ancestry (Fig. 2f ii). The contradiction could arise either from incorrect pedigree records of VTN 62–33-3 or a mislabeled genotype sample of VTN 62–33-3 or any of its ancestors. This caveat aside, it seems that VTN 62–33-3, along with Agria, was a major conduit for the *H1* locus into the European germplasm.

The second most frequent introgression, Cluster 11.1, included SNPs tagging the known late blight resistance locus *R3a/b* (Huang et al., 2005; Vos et al. 2015). The late blight resistance conferred by *R3a/b* was overcome soon after its deployment in varieties such as Pentland Dell (Malcolmson 1969). The persistence of this introgression at high frequency may be due to other resistance genes in linkage with this locus (Huang et al. 2004), quantitative resistance in combination with other resistance genes, or simply neutral processes following its rise to high frequency in elite breeding material (Fig. 4d).

For Cluster 11.1, we observed two major haplotypes amongst European cultivars, but we did not resolve their relationship to individual introgression donors, or differentiate introgressions from true *S. demissum* or the hybrid *S.* × *edinense.* Future work can address these questions about the haplotype diversity of the R3a/b resistance locus both within cultivated varieties and across *S. demissum* as a species.

We observed the cluster 11.1 introgressed alleles in an improved *S. tuberosum* Group Andigena cultivar, Diacol Capiro (DC). DC was produced by the Colombian Agricultural Institute (ICA) in 1968, and has been reported as a cross between Tuquerreña (CCC 61) and a potato named 1967 (CCC751) (‘DIACOL CAPIRO – Inventario de Tecnologías e Información para el Cultivo de Papa en Ecuador’, 2017). The most parsimonious explanation is that the introgression originally came from *S. demissum* into another variety, and was subsequently incorporated into Diacol Capiro as part of its improvement; perhaps through the ambiguous parent “1967”.

Cluster 8.1 was overall the most common VTN-62–33-3 derived haplotype. The conspicuous reduction in its frequency in the most recent set of Common Catalogue varieties (of which only one is a ‘starch’ potato, Fig. 4d) invites speculation that the locus under selection might be related to starch production. At least one study has mapped starch production QTLs to the south arm of Chromosome 8 (Schönhals et al. 2017), and this is supported by a recent GWAS study, which mapped a maturity phenotype (as a proxy for starch content) to this region (H. Li et al., 2024).

By integrating modern genomic data with historical breeding records, we traced the lineages through which key introgressions entered the European gene pool. For the two most widespread introgressions, we see a pattern of multiple independent introductions, but a rise to high frequency through a small number of major contributing ancestors. These results clarify how a limited number of breeding decisions shaped cultivated potato in Europe, complementing studies of its pre- and early European history (Sun et al. 2025; Zhang et al. 2025), and providing context for the diversity observed amongst modern European potato varieties.

## Materials and methods

### Identification of European varieties

We used the Common Catalogue of potato cultivars (Common Catalogue of varieties of agricultural plant species 2021), which are selected on the basis of: Distinctness, Uniformity, Stability, and Value for cultivation and use (where Value includes yield, disease resistance, and response to environment). We exclude from this definition those cultivars described as “conservation varieties”, which are of particular cultural or historical significance.

We selected all registered cultivars, along with those surrendered on or since 31st December 2020 (to include the last registered elite cultivars from the United Kingdom); this totalled 1706 unique cultivars, of which 1243 were named in the pedigree. We further removed 28 cultivars with unresolved duplications of the same name in the pedigree, and 6 cultivars that were known synonyms of other cultivars in the list; arriving at a list of 1209 varieties for analysis.

### Manual curation of pedigree information

There were 455 duplicate names shared between multiple varieties; in such cases, we assigned unique identifiers to each of the 1026 affected varieties. We then searched for pedigree records listing each variety as a parent, and used date and breeder information to assign the correct parent where possible (e.g. Ada [released 1962] = Saskia x Vera; There are two cultivars named Vera: VERA_17204 [1943] and VERA_17205 [1995], so we are able to resolve this parent as VERA_17204).

Where a duplicate name could not be resolved e.g. a cultivar might have a parent called White Rose with insufficient information to distinguish between White Rose (1871) and White Rose (1893), parentage was assigned to a dummy cultivar e.g. ‘WHITE ROSE_?’’.

We excluded any variety with “unknown”, “seedling” or “ x” in the name (the latter denoting an intermediate hybrid between two varieties).

### Major contributing ancestor (MCA) analysis of European varieties

We implemented a scoring algorithm following the basic assumption that parents contribute half of their genetic material to each of their offspring (Lansari et al. 1994; Love 1999; Sjulin & Dale 1987). Each European variety in the pedigree received a score of 1, and scores were propagated upwards through the pedigree, with each parent receiving an equal share of their child’s score. Thus, the score would accumulate at the cultivars having contributed the most to the European varieties (Fig. 1 a-c; Supplementary Fig. 1). Where duplicate variety names could not be resolved, the score was not propagated further. MCAs were then ranked in descending order of their contribution score. We considered varieties as being *S. vernei* if their name contained ‘S. vernei’ or its three-letter code ‘vrn’.

### Quantifying missing parentage in the pedigree

We split pedigree records into bins of 20 years. For each parent listed in the pedigree record, we counted any parent name that contained the strings ‘unknown’, ‘?’, ‘variety’ or ‘seedling’ as an unknown parent. Pedigree records where the parentage field was blank, we counted two unknown parents.

### Quantifying parentage by country of origin

A variety was defined as “European” if the pedigree record stated that it was released in a country on continental Europe, the British Isles, or Russia. “German” varieties included those originating from “BDR”, “DDR”, and “GER”. To avoid a confounding effect of breeding line records, and to focus the analysis on commercial cultivars, we excluded all varieties which contained digits in their names (with the exception of “7 FOUR 7”).

### Analysis of introgressed SNP clusters

We first checked the quality and position of SNP markers using the reference genome DM 1–3 516 R44 (v6.1) (Pham et al., 2020). We generated a BLASTN database (v2.14.1) (Camacho et al., 2009) of the reference genome using the makeblastdb command (-dbtype nucl), and queried it with the reference allele sequences (-max_target_seqs 5, -qcov_hsp_perc 90; Supplementary Data 2). We discarded any SNPs which had multiple perfect hits (pident > 99.999) or did not have any good hits (pident > 95). We then proceeded with 14259 SNPs.

We identified 47 pre-1945 varieties using the release dates from Vos et al. 2015. SNPs were considered monomorphic in these old varieties if all 47 varieties were 0-copy, or all were 4-copy (ignoring missing values). SNPs were then clustered on the first five principal components of their dosage across all 886 samples using HDBSCAN (with parameters min_samples = 5, min_clusters = 5, version as implemented by the scikit-learn python package v1.3.2) (McInnes et al., 2017), with missing values converted to a copy-number of 0. We took only the first 25 clusters for each chromosome. We considered a cluster ‘High confidence’ if any of the SNPs occurred in at least 5 European varieties (defined as a genotyped variety which was also a Common Catalogue variety).

For the analysis of haplotypes of the *H1* locus (cluster 5.1) and R3a/b locus (cluster 11.1/11.2), SNP dosage values were extracted as a SNP vs. genotype matrix and subjected to hierarchical clustering using Euclidean distance and average linkage (scipy.cluster.hierarchy.linkage). The R3a/b haplotype clusters were assigned using a fixed cluster number (k = 8; fcluster with criterion='maxclust').

Overlaps with QTLs reported in Gebhardt (2023) were determined by taking the bounding SNPs of each cluster and reporting any potato QTL which overlapped any part of the resulting region.

The location of STM1051 in the DM 1–3 516 R44 genome assembly (v6.1) was determined by taking the sequence of the forward primer TCCCCTTGGCATTTTCTTCTCC (Milbourne et al., 1998) and searching for homology using the BLAST tool provided by the SpudDB website (Hamilton et al., 2025).

### Major contributing ancestor (MCA) analysis of introgressed SNP clusters

When calculating the MCAs of a given SNP cluster: For each genotyped variety, we assigned it a score equal to the average dosage of derived alleles of SNPs in the cluster. We assigned a score of -1 to varieties which were genotyped with a dosage of 0 across all SNPs. The genotyped copy-number of a variety would not increase the score of the variety itself, but the score of its ancestors. When reporting independent pedigree lineages, we considered only the top 25 MCAs of a given SNP cluster.

We could additionally genotype the cultivar Semlo (parent of Agria) as containing at least 7/13 of the cluster 11.1 alleles using SNP array data from Selga et al. (2022) (Supplementary Table S8). This information was only used for the visualization of Fig. 3a (Selga et al., 2022).

### Analysis of introgression cluster occurrence over time

We manually matched 365 genotyped varieties with corresponding pedigree entries (Supplementary Table S5). Vos et al. (2015) noted that their ‘Urgenta’ sample was likely incorrectly named, so we removed it from further analysis. Since Urgenta was listed as a sample where many SNP-derived alleles were first observed, we recalculated which sample each SNP-derived allele was first observed in, taking the variety with the earliest year of release containing each allele.

We further subset the list of 365 genotyped pedigree varieties to include those which also occurred in the Common Catalogue, leaving 210 varieties (14 pre- and 196 post-1945). We binned these varieties by decade of release. Due to low numbers, we grouped all varieties released before 1945, and all varieties released between 1945 and 1970. For each introgression cluster, we asked how many of the varieties contained at least one of the introgressed alleles.

### Observation of SNPs in 11 Solanum vernei and 7 Andigena clones

Resequencing reads for samples of *Solanum vernei* and *Solanum tuberosum Group Andigena* were obtained from previous publications (Supplementary Table S10) (Hardigan et al., 2017; Kyriakidou et al., 2020; Y. Li et al. 2018; Tang et al., 2022; Zhang et al., 2019). Adapter trimming and quality filtering were conducted with Trim Galore (v0.6.10)(Krueger et al., 2023), requiring a minimum quality score of 20. Filtered reads were aligned to the *Solanum tuberosum* reference genome DM 1–3 516 R44 (v6.1)(Pham et al., 2020) using BWA-MEM (v0.7.17)(H. Li, 2013). Duplicates were marked using Picard Toolkit (“Picard Toolkit,” 2019). Mean coverage per chromosome was calculated using samtools depth, and alignment statistics such as percentage of reads mapped and insert size distribution were obtained using samtools (Danecek et al., 2021). Mapping quality and read depth distributions were further validated with Qualimap (v2.2.1) (Okonechnikov et al., 2016). Variant calling was performed using the Genome Analysis Toolkit (GATK, v4.2.0.0)(McKenna et al., 2010). Variant calling was conducted using HaplotypeCaller within GATK in GVCF mode, generating genomic variant call format (GVCF) files per sample. Joint genotyping was performed with GenotypeGVCFs within GATK, using a ploidy setting of 4. Variant filtration was applied with the following criteria: Missing data < 40%, Base Quality Rank Sum between − 2 and 2, Mapping Quality (MQ) > 45, MQ Rank Sum between -3 and 2, and Strand Odds Ratio (SOR) < 2. A SNP was scored as present in a sample if the sample was at least heterozygous for the expected alternate allele.

### Draft assembly of Solanum demissum

PacBio HiFi long sequencing reads were generated for a sample of *S. demissum* (Gross Lusewitz Potato Collections at the Leibniz Institute of Plant Genetics and Crop Plant Research (IPK); Accession number WKS30238. A contig-level assembly was performed using hifiasm v.0.19.6-r595 (Cheng et al., 2021), including HiFi (PacBio) and ultra-long (> 50 Kb) ONT Nanopore reads. Default parameters were used on the phasing mode –n-hap 6. The observed collapsed genome size is 2.2 Gb with a total of 5108 unitigs.

### Observation of clustered SNPs in assembled genomes

We downloaded diploid genome assemblies of 9 wild species closely related to *S. vernei* (Supplementary Table S10) from http://solomics.agis.org.cn/potato/ftp/genome/

## Supplementary Information

Below is the link to the electronic supplementary material.

Supplementary file1 (DOCX 6185 kb)

Supplementary file2 (TSV 639 kb)

Supplementary file3 (XLSX 61499 kb)

Supplementary file4 (XLSX 654 kb)

## Data Availability

Sequenced reads used for the assembly of *Solanum demissum* ‘El Desierto’ in this study have been deposited in the European Nucleotide Archive (ENA) at EMBL-EBI under accession number PRJEB101471. Supplementary Datasets and scripts used for the MCA and introgression analyses are available at https://github.com/schneebergerlab/PotatoMCAs.
