# Supplementary material for: Tracing modern breeding introgressions in European potato
Source: Theor Appl Genet. 2026 Feb 17;139(3):73. doi: 10.1007/s00122-025-05143-z (PMC12909365; doi:10.1007/s00122-025-05143-z)
Supplement: Supplementary file 1 — Supplementary file1 (DOCX 6185 kb) [file 122_2025_5143_MOESM1_ESM.docx]

**Supplementary Information**

**Supplementary Data 1: The curated Pedigree records as a tsv file**

**Supplementary Data 2: The SNP genotyping data reported in Vos et al. 2015.**

**Supplementary Tables:**

**Supplementary Table S1. The percentage of pedigree varieties with unknown parentage over time**

**Supplementary Table S2. Parentage of European Cultivars**

**Supplementary Table S3. Common Catalogue varieties in the pedigree**

**Supplementary Table S4. Major Contributing Ancestors of European Varieties**

**Supplementary Table S5. Genotyped varieties in pedigree**

**Supplementary Table S6. Clusters of post-1945 SNPs**

**Supplementary Table S7. Overlaps between SNPs introgressed via VTN 62-33-3, and population structure groups.**

**Supplementary Table S9. The Major contributing Ancestors of SNPs found in prominent modern introgression SNP clusters**

**Supplementary Table S10. List of public DNA samples used**

**Supplementary Table S11. SNPs detected in WGS samples of Solanum vernei**

**Supplementary Table S12. SNPs detected across WGS samples of *S.tuberosum* grp. Andigena, and *S.tuberosum* grp. Chilotanum**

**Supplementary Table S13. SNPs detected in genome assemblies of potato species**

**Supplementary Table S14. Heatmap of Cluster 5.1 haplotypes across genotyped varieties**

**Supplementary Table S15. Heatmap of Cluster 11.1&2 haplotypes across genotyped varieties**

**Supplementary Figures:**

**Supplementary Figure 1. Edge cases for Most Contributing Ancestor (MCA) analysis of elite European cultivars a)** When the parent of a cultivar cannot be resolved amongst multiple possible parents, then the score is propagated to a placeholder cultivar with the tag “_?”. The score accumulating in this placeholder cultivar are not propagated further up the pedigree. **b)** When the pedigree provides more than two parents, and does not indicate the order in which they were bred together, then the score of the child cultivar was distributed equally among them.

**Supplemental Figure 2. Two accounts for the ancestry of MPI 19268 from the pedigree.**

Red nodes are varieties in the pedigree. Question marks indicate unknown heritage. “dms x Hamburger Eier x Parnassia x Rotkargis x Deodara” is not resolved into nodes because the order of the crosses is unknown. Abbreviations: dms = *S.demissum*

**Supplementary Figure 3. SNP clusters on chromosome 1. a)** The first three principal components of the SNP matrix for this chromosome. Position along PC3 is shown by colour, **b)** The same plot is shown, now with coloured dots indicating which SNPs were clustered together. Each colour represents a distinct cluster. Black points indicate un-clustered SNPs, **c)** The physical position of these SNPs plotted along the chromosome. Green shading indicates the pericentromeric region. Height along the Y-axis shows how many genotyped European varieties contained each SNP.

**Supplementary Figure 4. SNP clusters on chromosome 2. a)** The first three principal components of the SNP matrix for this chromosome. Position along PC3 is shown by colour, **b)** The same plot is shown, now with coloured dots indicating which SNPs were clustered together. Each colour represents a distinct cluster. Black points indicate un-clustered SNPs, **c)** The physical position of these SNPs plotted along the chromosome. Green shading indicates the pericentromeric region. Height along the Y-axis shows how many genotyped European varieties contained each SNP.

**Supplementary Figure 5. SNP clusters on chromosome 3. a)** The first three principal components of the SNP matrix for this chromosome. Position along PC3 is shown by colour, **b)** The same plot is shown, now with coloured dots indicating which SNPs were clustered together. Each colour represents a distinct cluster. Black points indicate un-clustered SNPs, **c)** The physical position of these SNPs plotted along the chromosome. Green shading indicates the pericentromeric region. Height along the Y-axis shows how many genotyped European varieties contained each SNP.

** Supplementary Figure 6. SNP clusters on chromosome 4. a)** The first three principal components of the SNP matrix for this chromosome. Position along PC3 is shown by colour, **b)** The same plot is shown, now with coloured dots indicating which SNPs were clustered together. Each colour represents a distinct cluster. Black points indicate un-clustered SNPs, **c)** The physical position of these SNPs plotted along the chromosome. Green shading indicates the pericentromeric region. Height along the Y-axis shows how many genotyped European varieties contained each SNP.

**Supplementary Figure 7. SNP clusters on chromosome 5. a)** The first three principal components of the SNP matrix for this chromosome. Position along PC3 is shown by colour, **b)** The same plot is shown, now with coloured dots indicating which SNPs were clustered together. Each colour represents a distinct cluster. Black points indicate un-clustered SNPs, **c)** The physical position of these SNPs plotted along the chromosome. Green shading indicates the pericentromeric region. Height along the Y-axis shows how many genotyped European varieties contained each SNP.

** Supplementary Figure 8. SNP clusters on chromosome 6. a)** The first three principal components of the SNP matrix for this chromosome. Position along PC3 is shown by colour, **b)** The same plot is shown, now with coloured dots indicating which SNPs were clustered together. Each colour represents a distinct cluster. Black points indicate un-clustered SNPs, **c)** The physical position of these SNPs plotted along the chromosome. Green shading indicates the pericentromeric region. Height along the Y-axis shows how many genotyped European varieties contained each SNP.

** Supplementary Figure 9. SNP clusters on chromosome 7. a)** The first three principal components of the SNP matrix for this chromosome. Position along PC3 is shown by colour, **b)** The same plot is shown, now with coloured dots indicating which SNPs were clustered together. Each colour represents a distinct cluster. Black points indicate un-clustered SNPs, **c)** The physical position of these SNPs plotted along the chromosome. Green shading indicates the pericentromeric region. Height along the Y-axis shows how many genotyped European varieties contained each SNP.

** Supplementary Figure 10. SNP clusters on chromosome 8. a)** The first three principal components of the SNP matrix for this chromosome. Position along PC3 is shown by colour, **b)** The same plot is shown, now with coloured dots indicating which SNPs were clustered together. Each colour represents a distinct cluster. Black points indicate un-clustered SNPs, **c)** The physical position of these SNPs plotted along the chromosome. Green shading indicates the pericentromeric region. Height along the Y-axis shows how many genotyped European varieties contained each SNP.

** Supplementary Figure 11. SNP clusters on chromosome 9. a)** The first three principal components of the SNP matrix for this chromosome. Position along PC3 is shown by colour, **b)** The same plot is shown, now with coloured dots indicating which SNPs were clustered together. Each colour represents a distinct cluster. Black points indicate un-clustered SNPs, **c)** The physical position of these SNPs plotted along the chromosome. Green shading indicates the pericentromeric region. Height along the Y-axis shows how many genotyped European varieties contained each SNP.

** Supplementary Figure 12. SNP clusters on chromosome 10. a)** The first three principal components of the SNP matrix for this chromosome. Position along PC3 is shown by colour, **b)** The same plot is shown, now with coloured dots indicating which SNPs were clustered together. Each colour represents a distinct cluster. Black points indicate un-clustered SNPs, **c)** The physical position of these SNPs plotted along the chromosome. Green shading indicates the pericentromeric region. Height along the Y-axis shows how many genotyped European varieties contained each SNP.

** Supplementary Figure 13. SNP clusters on chromosome 11. a)** The first three principal components of the SNP matrix for this chromosome. Position along PC3 is shown by colour, **b)** The same plot is shown, now with coloured dots indicating which SNPs were clustered together. Each colour represents a distinct cluster. Black points indicate un-clustered SNPs, **c)** The physical position of these SNPs plotted along the chromosome. Green shading indicates the pericentromeric region. Height along the Y-axis shows how many genotyped European varieties contained each SNP.

** Supplementary Figure 14. SNP clusters on chromosome 12. a)** The first three principal components of the SNP matrix for this chromosome. Position along PC3 is shown by colour, **b)** The same plot is shown, now with coloured dots indicating which SNPs were clustered together. Each colour represents a distinct cluster. Black points indicate un-clustered SNPs, **c)** The physical position of these SNPs plotted along the chromosome. Green shading indicates the pericentromeric region. Height along the Y-axis shows how many genotyped European varieties contained each SNP.

**Supplementary Figure 15. Extended Pedigree tracing of the top 25 MCAs for Cluster 8.1**

**a)** Table of scores from Major Contributing Ancestor analysis using the genotype of Cluster 8.1 SNPs. AM breeding lines from the *Stitchung voor Plantenveredling* are identified as key contributors of the 10 SNPs. *Genotype* is the average copy number of the 10 clustered SNP derived alleles, if known. *#Offspring* is the number of direct offspring in the pedigree, *Score* is the Contribution Score resulting from the MCA algorithm. **b-c)** Interrelationships of the top 25 contributing ancestors of Chr08 cluster 1 SNPs. AM 78-3736, can be inferred to also contain the variants; given that both of its offspring with KA 77-0133 (Kartel and Karakter) contain the SNPs, and KA 77-0133 itself does not. Node colour indicates the Contribution Score. Grey nodes are unnamed hybrids. Hyphens in nodes indicate which of the displayed samples were genotyped. Black block arrows show the contributions of two possible *Solanum vernei* ancestors. Insets (grey box): additional VTN 62-33-3 lineages separated for visual simplicity. Abbreviations: vrn = *S. vernei, spg = S. spegazzinii, opl= S. oplocense,* lpt=*S, leptostigma*, sct=*S. sanctae-rosae,* phu = *S. tuberosum* Group Phureja

**Supplementary Figure 16. Extended pedigree tracing of MCAs for introgressed SNP cluster 5.1 a)** Black block arrows indicate likely introgression donors. Node colour indicates the Contribution Score. Grey nodes are unnamed hybrids. +/- in nodes indicate which of the displayed samples were genotyped, plus indicating that at least one derived allele was present. Inset top right: Additional contributing ancestors, not connected to the other three groups. Abbreviations: adg = *S. tuberosum* Group Andigena, dms = *S. demissum,* vrn = *S. vernei*

**Supplementary Figure 17. Haplotype clustering of Cluster 5.1**

Genotyped varieties are aligned along the y-axis. A dendrogram on the far left shows clustering of the haplotypes. The purple heatmap shows the allele dosage of the derived allele for each SNP.

**Supplementary Figure 18. Extended Pedigree tracing of the Top 25 MCAs for Introgressed SNP Cluster 11.1.** Node colour indicates the Contribution Score. Grey nodes are unnamed hybrids. +/- in nodes indicate which of the displayed samples were genotyped: plus indicating that at least one derived allele was present. Black block arrows indicate likely introgression donors. Inset bottom: Additional contributing ancestors, not obviously connected to the other three groups. Abbreviations: vrn = *S. vernei,* dms = *S. demissum,* phu = *S. tuberosum* Group Phureja, sto= *S. stoloniferum*

**
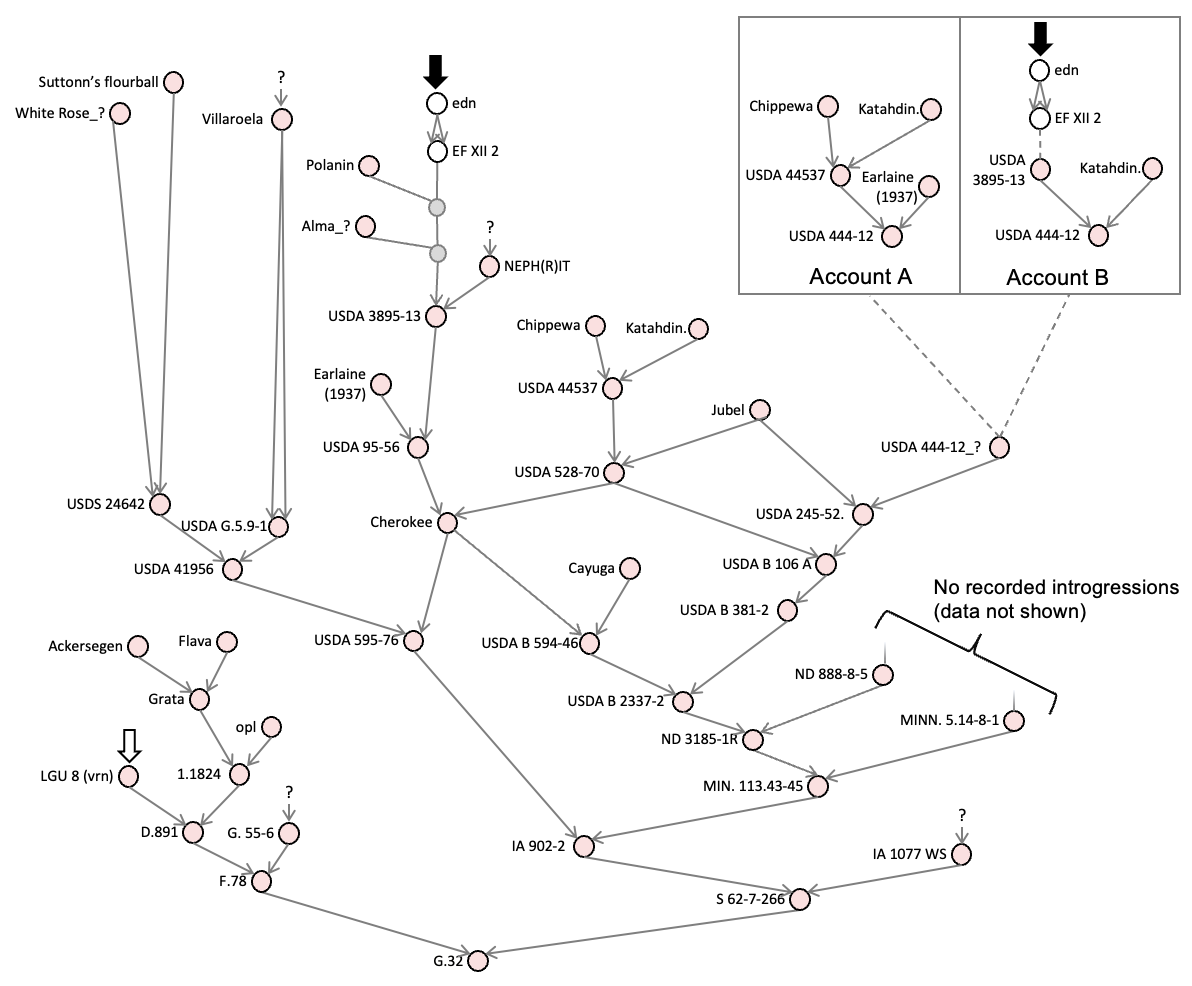
**

**Supplementary Figure 19. The pedigree of G.32 reveals two possible introgressions from *S. × edinense***

Nodes indicate varieties, grey arrows indicate direction of inheritance. Black and white arrows show possible introgressions. Inset top right: The pedigree database gives two accounts of the pedigree of USDA 444-12, one includes *S. × edinense* the other does not. Abbreviations: edn = *S. ×* *edinense*

**Supplementary Figure 20. Introgression SNPs in genome assemblies*.*** A phylogenetic tree showing *S. vernei* and its most closely related neighbours within a subclade of wild potato relatives, adapted from Tang et al. (2022) (left). **a)** Presence/absence of Cluster 8.1 SNP derived alleles in *S. vernei* (4 out of 10) and related species. Genomes of *S. demissum*, and three genomes of *S. tuberosum* Group Andigena (including the improved cultivar DC) are shown beneath. **b)** Presence/absence of Cluster 5.1 SNP derived alleles. **c)** Presence/absence of Cluster 11.1 SNPs derived alleles. Some SNPs could not be observed due to poor alignment or deletions spanning the SNP location (grey shading).
